# Huge intermediate-grade myofibroblastic sarcoma in the retroperitoneum revealed by ^18^F-FDG PET/CT: a case report

**DOI:** 10.3389/fmed.2024.1461749

**Published:** 2024-09-12

**Authors:** Xiaotian Li, Tengyue Mei, Pan Wang, Jiong Cai, Xianwen Hu

**Affiliations:** ^1^Department of Nuclear Medicine, Affiliated Hospital of Zunyi Medical University, Zunyi, China; ^2^Department of Nuclear Medicine, People's Hospital of Qianxinan Buyi and Miao Minority Autonomous Prefecture, Xingyi, China

**Keywords:** myofibroblastic sarcoma, 18F-FDG, PET/CT, computed tomography, retroperitoneal

## Abstract

Myofibroblastic sarcoma (MS) is a relatively rare malignant bone and soft tissue tumor, which originates from myofibroblasts, with some characteristics of both smooth muscle cells and fibroblasts. It can develop in individuals at any age and can affect various regions, especially the head and neck; however, it is rarely reported retroperitoneally. Generally, this type of sarcoma is considered a low-grade malignancy, and cases classified as moderate and high-grade malignancy are rare. In this study, we describe a case of intermediate-grade myofibroblastic sarcoma (IGMS) originating from the retroperitoneum, which was confirmed through pathological diagnosis. The ^18^F-fluorodeoxyglucose (^18^F-FDG) positron emission tomography (PET)/computed tomography (CT) scan revealed a large, borderless mass located retroperitoneally with a significantly increased ^18^F-FDG uptake, accompanied by adjacent visceral and soft tissue infiltration and peripheral lymph node metastasis. The patient received chemotherapy for 3 weeks; however, the tumor did not shrink significantly. Therefore, the patient discontinued the treatment. After 5 months, his condition gradually deteriorated, which eventually led to death. Through this case report, the diagnosis and treatment of moderate malignant retroperitoneal myofibroblastoma were discussed, aiming to increase clinicians' understanding of this disease.

## Introduction

Myofibroblastic sarcoma (MS) is a relatively rare malignant bone and soft tissue tumor of myofibroblastic origin with some characteristics of both smooth muscle cells and fibroblasts (1). It was first reported by Professor Mentzel et al. (2) in 1998. MS is a controversial tumor, and there has been a lack of clear and uniform diagnostic criteria (3). Until 2002, the World Health Organization (WHO) classified MS as a new disease and a rare tumor in the classification of soft tissue tumors; however, its classification only lists it as low-grade, and there is no consensus on the definition of intermediate-grade and high-grade MS (4). MS can develop in individuals at any age and can affect various regions. Its incidence is difficult to calculate due to its diverse manifestations and the locations where it can occur. The imaging findings of MS are related to the tissue composition of the lesion, often showing round or fusiform soft tissue masses with unclear boundaries, and liquefaction necrosis is also commonly observed (5, 6). Currently, most reported cases of MS are generally classified as low-grade malignancies, while moderate and highly malignant cases are rare. The clinical presentation of MS in patients is non-specific, typically manifesting as painless, progressive, and enlarged masses (7). In this study, we present a rare case of an intermediate-grade myofibroblastic sarcoma (IGMS), located in the retroperitoneal region, which was confirmed through pathological diagnosis. In the early stage of the disease, the patient presented with left waist pain without an obvious cause, and the computed tomography (CT) examination showed severe left kidney hydrosis with an unclear kidney contour. The patient was diagnosed with a left kidney abscess with sinus formation in a local community hospital and treated with antibiotics for 2 weeks; however, no improvement was observed. Then, he visited our hospital for further diagnosis and treatment. The ^18^F-fluorodeoxyglucose (^18^F-FDG) positron emission tomography (PET)/CT scan showed a large, borderless mass located retroperitoneally with a significantly increased radiological uptake, accompanied by adjacent visceral and soft tissue infiltration and peripheral lymph node metastasis. The patient received chemotherapy for 3 weeks; however, the tumor did not shrink significantly. Through this case report, the clinical diagnosis and treatment of IGMS were discussed, aiming to increase clinicians' awareness of the disease.

## Case description

**Figure 1:**
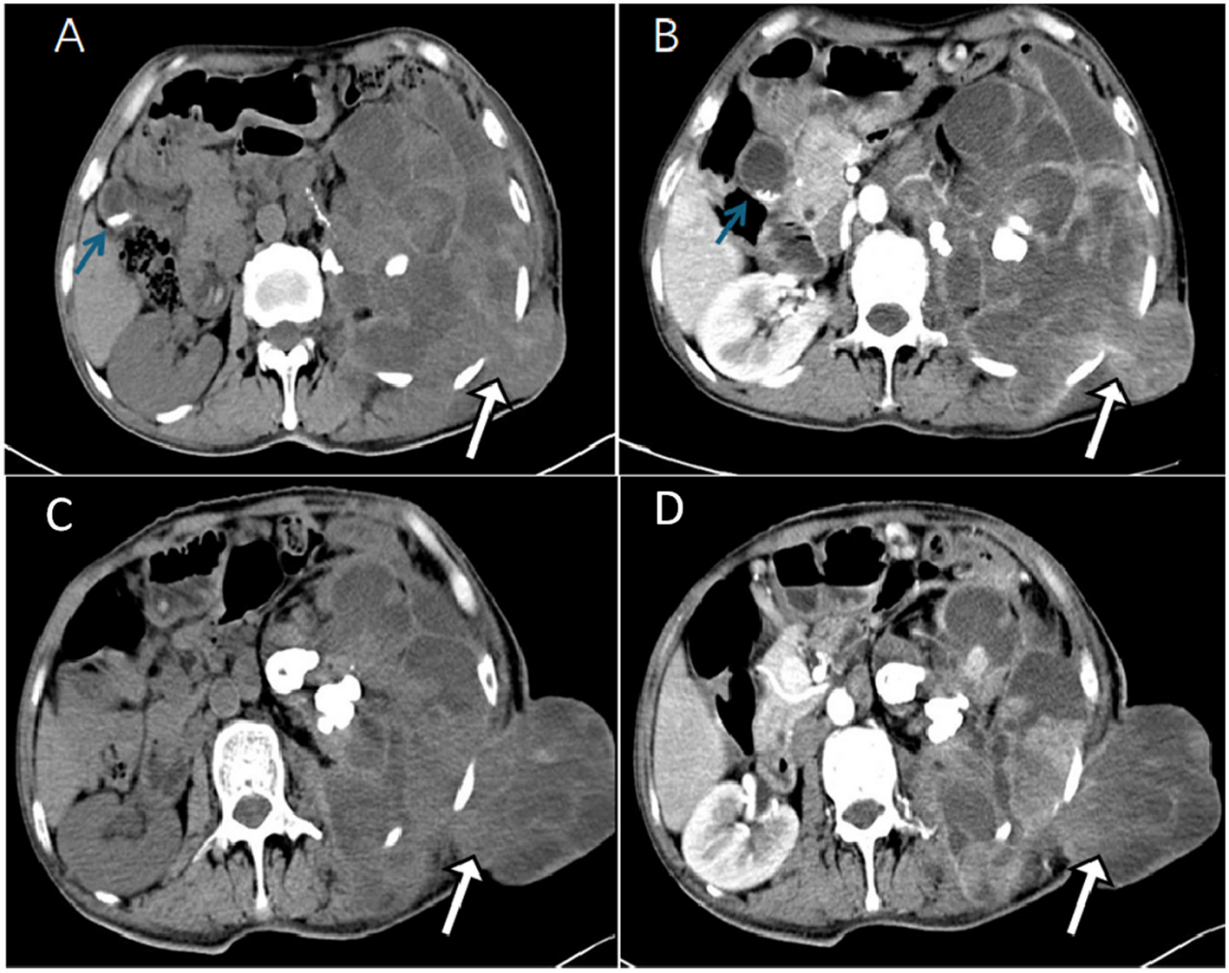
The computed tomography (CT) plain scan of the abdomen in a local community hospital in August 2023 showed a large mixed density image in the retroperitoneal space around the left kidney, with lesions protruding from the left abdominal wall at a maximum cross-section of 5.0 cm × 4.2 cm [**(A)**, white arrow]. The lesions showed uneven moderate enhancements in the arterial phase **(B)**. In addition, high density lithiasis was observed in the gallbladder area [**(A, B)** blue arrow]. A month later, the patient was treated in Zunyi Medical University. The abdominal CT plain scan showed a large mixed density shadow in the retroperitoneal space around the left kidney and prominent lesions in the left abdominal wall with a maximum cross-section of approximately 10.7 cm × 8.2 cm [**(C)**, white arrow], which was significantly larger than the previous one. The lesions showed mild to moderate enhancements in the arterial phase **(D)**.

**Figure 2:**
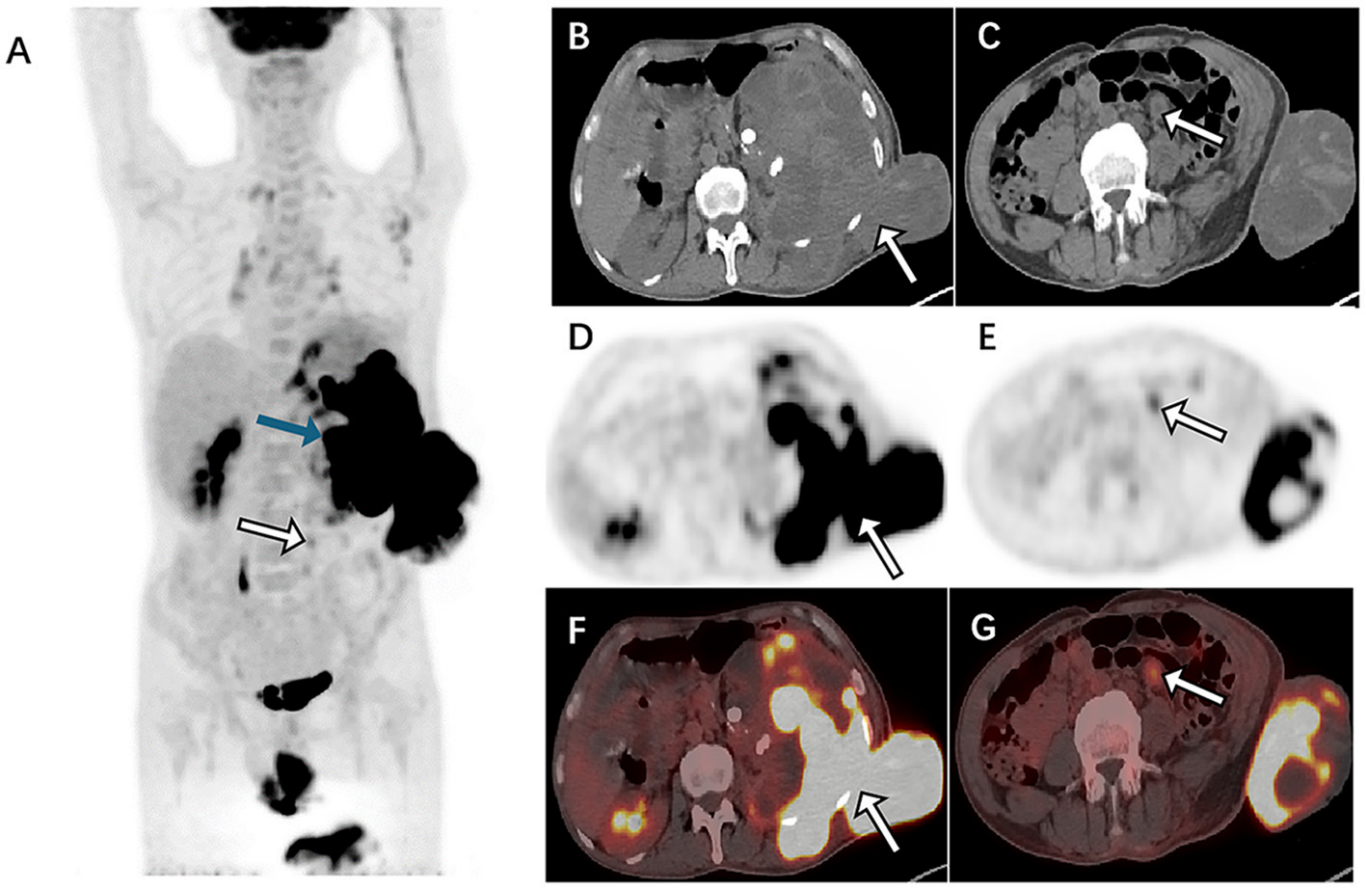
^18^F-FDG PET/CT **(A)** Maximum intensity projection (MIP) was subsequently performed to further evaluate the patient's whole-body condition, and it revealed an increased uptake of radioactivity in large patches of the left abdomen (blue arrow) and one of the high metabolic non-specific lymph node uptakes (white arrow). The axial image CT **(B)** showed the retroperitoneal left perirenal space and highlighted an increased uptake of fluoro-18 fluorodeoxyglucose (^18^F-FDG) in the left abdominal wall (SUVmax30.1). [**(B)**: CT, **(D)**: PET, **(F)**: fusion]. The images [**(C)**: CT, **(E)**: PET, **(G)**: fusion] showed abdominal lymph nodes (black arrows), approximately 1.4 cm × 1.2 cm in size, with partial necrotic lesions and abnormal metabolic fusion, and SUVmax was 1.9.

A 49-year-old man with unexplained left lumbar pain and a left lumbar skin abscess presented to a local community hospital in August 2023 and was initially diagnosed with a left kidney abscess with sinus formation. Then, he received treatment that included a puncture and placement of an indentation catheter in the left kidney, an incision to drain an abscess, and an antibiotic intravenous infusion for 2 weeks. Despite these interventions, there was no significant improvement in his symptoms. For further diagnosis and treatment, he was admitted to our hospital in November 2023. A physical examination revealed a large area of ulcers on his left waist skin, and a lump of approximately 10 × 12 cm could be seen locally protruding from the skin surface, showing signs of ulceration and bleeding on the surface of the lump. Below the mass, there is a sinus tract with purulent discharge. The laboratory examination results showed that the white blood cell (WBC) count, neutrophil absolute value (NEUT#), monocyte absolute value (MON#), basophil absolute value (BASO#), total bile acid (TBA), hypersensitive C-reactive protein (hsCRP), and endogenous creatinine clearance rate (ccr) level increased, while the hemoglobin, albumin, sodium, and chlorine values decreased. The remaining laboratory indicators were not abnormal. The results of the above laboratory tests are presented in Table 1. The abdominal CT examination before and after intravenous contrast media containing iodine (Figure 1) showed that the contour of the left kidney was not clear, and the surrounding space of the left kidney showed a large mixed density shadow, gas accumulation, and protrusion from the left abdominal wall; multiple lymph node enlargements could be seen in the abdominal cavity. Based on these imaging findings, we initially considered it to be a neoplastic lesion. The patient refused undergoing magnetic resonance imaging (MRI) due to claustrophobia. Instead, he underwent a whole-body PET/CT examination to evaluate the nature of the lesion and determine the best course of treatment. The results showed that there was a large, flaked, increased ^18^F-FDG uptake in the retroperitoneum, and the lesion was extensive and protruded outward through the left abdominal wall. Moreover, multiple enlarged lymph nodes with an increased ^18^F-FDG uptake in the abdominal cavity, mediastinum, and left axillary lymph nodes were also observed (Figure 2). The above presentation suggested a retroperitoneal origin of the malignant tumor invading the adjacent tissues with multiple metastases. During that time, the patient was in a poor and critical condition with large tumor invasion and rapid disease progression. After consulting a multidisciplinary tumor committee, which included radiologists, urologists, vascular surgeons, and anesthesiologists, we decided to explore the nature of the tumor without considering surgical resection. Subsequently, the patient underwent a partial removal of the tumor that had penetrated through the skin on the left lower back for a histopathological examination.

**Table 1:** Laboratory examination and tumor marker test results.

**Index**	**Value**	**Unit**	**Annotation**	**Reference**
WBC	12.36 × 10^−9^	/L	Up	(4–10) × 10^−9^
NEUT#	9.64 × 10^−9^	/L	Up	(1.8–6.3) × 10^−9^
MON#	1.36 × 10^−9^	/L	Up	(0.1–0.6) × 10^−9^
BASO#	0.12 × 10^−9^	/L	Up	(0–0.06) × 10^−9^
TBA	10.70	μmol/L	Up	0.14–9.66
hsCRP	162.72	mg/L	Up	0.068–8.200
Ccr	74.16	ml/min	Down	80–120
HB	105	g/L	Down	130–175
albumin	22.9	g/L	Down	40–55
sodium	128.5	mmol/L	Down	137–147
chlorine	95.7	mmol/L	Down	99–110

WBC, white blood cell; NEUT#, neutrophil absolute value; MON#, monocyte absolute value; BASO#, basophil absolute value; TBA, total bile acid; hsCRP, Hypersensitive C-reactive protein; Ccr, endogenous creatinine clearance rate; HB, hemoglobin.

**Figure 3:**
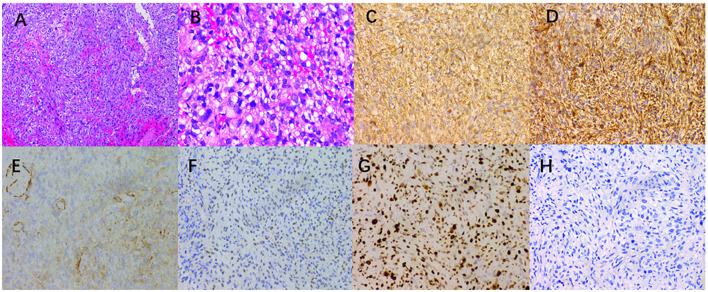
**(A)** A large number of spindle cells, a large number of small thin-walled blood vessels in the tumor cells, and inflammatory cell infiltration were observed in the interstitium (hematoxylin-eosin × 100). **(B)** Hematoxylin-eosin (magnification, × 400) showed that the mitotic images were rare. The immunohistochemical images showed that the tumor positively expressed SMA **(C)**, vimentin **(D)**, calponin **(E)**, WT-1 **(F)**, Ki-67 [**(G)**, index 70%], and negative desmin **(H)** ( × 200).

Under the microscope, the tumor tissue appeared grayish-red, with an abundance of fusiform cell proliferative lesions (Figure 3). The immunohistochemical results revealed that the tumor cells positively expressed smooth muscle actin (SMA), vimentin, CD34, S-100, cytokeratin (CK), calponin, WT-1, P63, CK7, H3K27me3, and Ki-67, with a positive index of approximately 70%, while these cells negatively expressed CD117, desmin, PAX-8, TFE3, and GATA3. Based on the pathology and immunohistochemical results, the patient was diagnosed with IGMS. The patient received chemotherapy for 3 weeks; however, the tumor did not shrink significantly. Therefore, the patient refused further treatment. After 5 months, the patient's condition gradually deteriorated, which eventually led to death.

## Discussion

MS can develop in individuals at any age and can affect different sites, most commonly the head and neck and especially the mouth, bones, trunk, limbs, and other sites, but it is rarely reported retroperitoneally. By reviewing the literature, we found that only three cases of malignant retroperitoneal MS have been reported, including one case of low-grade malignancy (8), one case of intermediate-grade malignancy (9), and one case of high-grade malignancy (10). The clinical features of MS are related to the size, location, and growth pattern of the tumor. Patients often present with the symptoms of tumor compression, and some patients only present with a painless progressive enlargement of the mass (5). The retroperitoneal MS, like most retroperitoneal tumors, has no obvious clinical symptoms. A few patients present with abdominal pain, abdominal distention, or palpable abdominal mass and gastrointestinal compression symptoms, such as abdominal distension, loss of appetite, and other non-specific clinical manifestations (9, 10). The patient who we reported was a 49-year-old man who had no clinical symptoms at the early stage but later developed progressive abdominal pain symptoms with short-term aggravation, an abdominal mass that broke through the skin of his left waist, and gastrointestinal compression symptoms, including abdominal distension symptoms, which were consistent with those reported in the literature for MS.

In terms of imaging, low-grade myofibroblastic sarcoma (LGMS) can be observed on CT scans as tumors with either homogeneous or heterogeneous medium-density tumors. Most of these tumors have clear boundaries and contain large flake-like calcifications (11). The imaging findings of IGMS are related to the tissue composition of the lesion (6). CT often shows round or fusiform soft tissue masses with unclear boundaries, mixed tissue density, common liquefaction necrosis, and scattered calcification in some tumor bodies (12). The lesions are invasive and often spread to the surrounding fat, glands, muscle, and skeletal tissue (13). The enhancement mode of an MS-enhanced scan is related to the amount of fiber components in the lesion, and the enhancement mode is varied, often showing mild uneven enhancements (14, 15). Moreover, some scholars suggest that the enhancement mode of a tumor may be related to the rich microvascular density and capillary permeability of tumor mesenchyma and the degree of tumor myxosis (5, 16). In this case, the lesion showed a mildly uneven enhancement in the arterial phase, which may be related to more fiber components and insufficient blood supply at the lesion edge. However, CT has shortcomings in diagnosing IGMS. As IGMS has unclear boundaries and is more aggressive, in this case, the space between the left kidney and retroperitoneum, as well as left kidney hydrops and abnormal morphology, could not be clearly distinguished during the CT, so it was initially misdiagnosed as a left kidney abscess with sinus formation for treatment. During the PET/CT scan, an irregularly uniform retroperitoneal lesion with a significantly increased ^18^F-FDG uptake was seen, and multiple lymph node metastases with an increased ^18^F-FDG uptake were also detected at the same time, suggesting that ^18^F-FDG PET/CT plays an important role in the diagnosis of IGMS.

It is generally accepted that MS is usually a low-grade malignancy with a higher risk of recurrence and a lower incidence of metastasis (7). However, our case study showed that the patient with IGMS had a higher incidence of metastasis, and this condition was highly invasive, aggravating the spread to neighboring tissues and lymph nodes and causing distant metastasis. The symptoms of systemic lymph node enlargement are well distinguished between IGMS and typical lymphoma. By further reviewing the literature (17, 18), the most significant differences between MS and typical lymphoma are that, on the one hand, in lymphoma, the density of the lymph nodes involved is generally uniform, the distribution is mostly symmetrical, and there are usually many lymph nodes fused with each other to form large lymph nodes. On the other hand, in MS, the density of the lymph nodes involved is not uniform, there is obvious liquefaction necrosis, the distribution is not symmetrical, and there is no fusion phenomenon in the larger lymph nodes involved. The patient we reported had uneven density of the enlarged lymph nodes, with obvious liquefaction necrosis and asymmetric distribution. This was consistent with the reports in the literature.

The final diagnosis of MS is determined by histopathology, and MS has different immunophenotypes. Histologically, MS is composed of spindle-shaped cells arranged in interwoven bundles, and the tumor cells are rich in rough endoplasmic reticulum and filaments (19). In immunohistochemistry, SMA, muscle-specific actin, and fibronectin are positively expressed in most cases, while desmin is positively expressed only in a few cases (19, 20). A large number of spindle-shaped cells, arranged in interwoven bundles, were observed under the microscope after examining the lesion section of the patient. In terms of immunohistochemistry, SMA, vimentin, CD34, S-100, CK, calponin, WT-1, P63, CK7, and H3K27me3 were all positive, which was consistent with the pathological diagnosis of MS.

Currently, the most widely used method for classifying tumor malignancy is the histological and pathological classification method of soft tissue tumors developed by the French Federation of Cancer Centers Sarcoma Group (FNCLCC) (19). The score is based on the degree of tumor differentiation, the number of mitosis per 10 high-power fields (10HPF), and tumor necrosis. The total score of 2–3 is classified as low grade, 4–5 as intermediate grade, and 6–9 as high grade (21, 22). In addition, it has been suggested that the Ki-67 labeling index may be helpful for MS classification as it can be used to evaluate the proliferative activity of tumor cells. The Ki-67 index of high-grade MS is higher than that of low-grade MS (15, 23). In our study, according to the FNCLCC grading, the score of the patient was 4, which consisted of 2 points for the degree of differentiation, 1 point for every 10HPF <9 mitosis, and 1 point for <50% tumor necrosis. Additionally, the Ki-67 index was approximately 70%, which is consistent with IGMS.

IGMS treatment usually involves surgical removal, radiation, and chemotherapy, depending on the location, size, and grade of the tumor, as well as the patient's overall health. Compared with LGMS, IGMS and high-grade malignant MS have obvious pleomorphism, mitotic activity, local necrosis, and significant invasiveness, with a higher local recurrence and metastasis. In two published studies with larger sample sizes, the recurrence rate of MS was 18% (2) and 54% (24), the latter of which included IGMS. Currently, the most effective method is complete resection, so the surgery should aim for R0 resection, which includes the removal of the entire tumor along with adjacent organs (25, 26). The need for adjuvant chemotherapy and radiotherapy is currently under debate (27, 28). However, in the case of retroperitoneal tumors, which are often large, affect multiple organs, and have a complex anatomy, there is limited literature on systemic chemotherapy after surgery, which emphasizes the need for multidisciplinary collaboration before and during surgery. Surgical resection combined with adjuvant radiotherapy and chemotherapy is considered to be the ideal treatment choice (9, 10, 28).

## Conclusion

IGMS is associated with a higher incidence of metastasis, and the absence of any therapeutic intervention suggests that survival rates are significantly low in its natural history. ^18^F-FDG PET/CT can help provide an accurate preoperative evaluation, effectively detect hidden lesions and distant metastases, and formulate reasonable treatment plans.

## Data Availability

The original contributions presented in the study are included in the article/supplementary material, further inquiries can be directed to the corresponding authors.
